# Effect of multifunctional cationic polymer coatings on mitigation of broad microbial pathogens

**DOI:** 10.1128/spectrum.04097-23

**Published:** 2024-08-05

**Authors:** Jianliang Gong, Chun-Yin Or, Eric Tung-Po Sze, Sidney Man-Ngai Chan, Pak-Long Wu, Peggy Miu-Yee Poon, Anthony K. Y. Law, Lucie Ulrychová, Jan Hodek, Jan Weber, Hui Ouyang, My Yang, Stephanie M. Eilts, Montserrat Torremorell, Yaakov Knobloch, Christopher J. Hogan, Christine Atallah, Juliette Davies, John Winkler, Ryan Gordon, Reza Zarghanishiraz, Mojtaba Zabihi, Cole Christianson, Deanne Taylor, Alan Rabinowitz, Jared Baylis, Joshua Brinkerhoff, Jonathan P. Little, Ri Li, Jeanne Moldenhauer, Michael K. Mansour

**Affiliations:** 1 C-POLAR Technologies Inc., West Vancouver, British Columbia, Canada; 2 Department of Chemistry, The Chinese University of Hong Kong, Hong Kong, China; 3 School of Science and Technology, Hong Kong Metropolitan University, Hong Kong, China; 4 Department of Mechanical Engineering, Hong Kong Polytechnic University, Hong Kong, China; 5 Institute of Organic Chemistry and Biochemistry of the Czech Academy of Sciences, Prague, Czechia; 6 Department of Genetics and Microbiology, Charles University, Faculty of Sciences, Prague, Czechia; 7 Department of Mechanical Engineering, University of Minnesota, Minneapolis, Minnesota, USA; 8 Department of Mechanical Engineering University of Texas-Dallas, Richardson, Texas, USA; 9 Department of Veterinary Population Medicine, University of Minnesota, Saint Paul, Minnesota, USA; 10 Division of Infectious Diseases, Massachusetts General Hospital, Boston, Massachusetts, USA; 11 School of Health and Exercise Sciences, University of British Columbia, Kelowna, British Columbia, Canada; 12 School of Engineering, University of British Columbia, Kelowna, British Columbia, Canada; 13 School of Nursing, University of British Columbia, Kelowna, British Columbia, Canada; 14 Interior Health Authority, Kelowna, British Columbia, Canada; 15 Rural Coordination Center of British Columbia, Vancouver, British Columbia, Canada; 16 Faculty of Medicine, University of British Columbia, Vancouver, British Columbia, Canada; 17 St. Paul’s Hospital, Vancouver, British Columbia, Canada; 18 Department of Emergency Medicine, University of British Columbia, Vancouver, British Columbia, Canada; 19 C-POLAR Technologies, Inc., Las Vegas, Nevada, USA; 20 Department of Medicine, Harvard Medical School, Boston, Massachusetts, USA; Taichung Veterans General Hospital, Taichung, Taiwan

**Keywords:** cationic polymer, SARS-CoV-2, Gram-positive, Gram-negative

## Abstract

Infection control measures to prevent viral and bacterial infection spread are critical to maintaining a healthy environment. Pathogens such as viruses and pyogenic bacteria can cause infectious complications. Viruses such as SARS-CoV-2 are known to spread through the aerosol route and on fomite surfaces, lasting for a prolonged time in the environment. Developing technologies to mitigate the spread of pathogens through airborne routes and on surfaces is critical, especially for patients at high risk for infectious complications. Multifunctional coatings with a broad capacity to bind pathogens that result in inactivation can disrupt infectious spread through aerosol and inanimate surface spread. This study uses C-POLAR, a proprietary cationic, polyamine, organic polymer with a charged, dielectric property coated onto air filtration material and textiles. Using both SARS-CoV-2 live viral particles and bovine coronavirus models, C-POLAR-treated material shows a dramatic 2-log reduction in circulating viral inoculum. This reduction is consistent in a static room model, indicating simple airflow through a static C-POLAR hanging can capture significant airborne particles. Finally, Gram-positive and Gram-negative bacteria are applied to C-POLAR textiles using a viability indicator to demonstrate eradication on fomite surfaces. These data suggest that a cationic polymer surface can capture and eradicate human pathogens, potentially interrupting the infectious spread for a more resilient environment.

**IMPORTANCE:**

Infection control is critical for maintaining a healthy home, work, and hospital environment. We test a cationic polymer capable of capturing and eradicating viral and bacterial pathogens by applying the polymer to the air filtration material and textiles. The data suggest that the simple addition of cationic material can result in the improvement of an infectious resilient environment against viral and bacterial pathogens.

## INTRODUCTION

Viruses and pathogenic bacteria can cause a broad range of infectious complications, including skin soft tissue infections caused by *Staphylococcus aureus* and respiratory infections, such as those caused by SARS-CoV-2 (COVID-19) and influenza virus. While evidence shows that many respiratory pathogens are spread through the aerosol route (1–6), pathogens are also found on surfaces (7–10) and in water (9, 11). Many pathogens can remain infectious for a prolonged time in the environment. Developing technologies to mitigate the spread of pathogens through airborne routes, surfaces, and water application is needed, especially for patients with compromised immunity or comorbidities that leave them at risk for infection.

Multifunctional coatings with broad application for pathogen binding and inactivation in aerosol, inanimate surfaces, and liquid would be ideal (11–13). One tractable route for material augmentation using a multifunctional coating is the application of cationic polymers (14–20).

C-POLAR is a proprietary cationic, polyamine, organic polymer with several key advantages, including being a charged, dielectric material generating both internal and external electric fields. In aqueous suspensions, the highly positive charge density polymers can aid in capturing, binding, and inactivating microorganisms and viruses (21, 22). Given the net negative charge of many pathogens, cationic polymers have displayed biocidal properties, including the inactivation of wild-type, drug-resistant human and avian influenza viruses and pathogenic bacteria such as *Escherichia coli* and *S. aureus* (15, 23, 24).

However, cationic polymers have not been applied to air filtration, specifically onto filters and fiber composites for aerosol or liquid pathogen collection and inactivation. To better define the application of C-POLAR to aerosol inactivation, we investigated the capacity of C-POLAR-coated filter material to bind and inactivate viral particles, including viable SARS-CoV-2 and Gram-positive and Gram-negative pathogenic bacteria.

## MATERIALS AND METHODS

### General preparation of C-POLAR-textiles

The C-POLAR-coated material (C-POLAR Technologies, Las Vegas, Nevada) is comprised of a hydrophobic layer and an electret layer. The hydrophobic base layer is a polyester nonwoven spun lace structure. The electret layer is comprised of the C-POLAR cationic polymer. The manufacturing of C-POLAR uses the deposition of cationic material onto the spunlace fabrics on a collection belt to deliver a uniform bonding of C-POLAR to fibers. The fibers are separated during the web-laying process by air jets. To bond C-POLAR to the polyester, heat is applied to partially melt the polymer, fusing the fibers. Textiles with solutions composed of 4% and 6% C-POLAR were used.

### Preparation of controls and C-POLAR-treated samples

The samples were prepared using either spunlace composed of 40 g or 145 g nonwoven spunlace consisting of 50% polyester/50% cotton. Control (untreated control), 4%, 6%, and 8% C-POLAR-treated fabric were generated. Test samples were coated with C-POLAR and then washed for 45 s in a water bath and dried in preparation for biological studies.

### Preparation of viable SARS-CoV-2 virus

SARS-CoV-2 (strain hCoV-19/Czech Republic/NRL_6632_2/2020) was isolated in a biosafety level 3 laboratory from a nasopharyngeal swab and propagated in Vero E6 cells (ATCC, Manassas, VA). The fourth passage was used for experiments. All work with the SARS-CoV-2 virus was performed in the Biosafety Level 3 laboratory per the permit issued by the State Office for Nuclear Safety, Department of Non-Proliferation, Chemical, and Biological Weapons Prohibition Division (permit number SÚJB/OKZCHBZ/6186/2020).

### Determination of biocompatibility and cytotoxicity of C-POLAR treated filter

To determine toxicity to Vero cells, control, and 4%, 6%, and 8% C-POLAR-coated 40 g nonwoven spunlace were incubated in triplicate in 10 mL of Dulbecco’s modified Eagle’s medium (DMEM) with 10% of fetal bovine serum (FBS) for 30 min. The wash-out solution was serially 10-fold diluted and transferred to Vero E6 cells (ATCC) in triplicate, which were seeded 24 h before. After 3 days of incubation at 37°C, 5% CO_2_, the cell viability was determined by the addition of XTT solution (50:1 mixture of 1 mg/mL XTT labeling reagent and 0.383 mg/mL PMS electron-coupling reagent) to the wells and incubated for 4 h at 37°C, 5% CO_2_. The absorbance of newly formed orange formazan dye was measured in an EnVision plate reader (PerkinElmer, Waltham, MA) at 450 nm. The absorbance was normalized to the absorbance of no sample control set to 100% and plotted versus log_10_ dilution using GraphPad Prism v.9.4 (GraphPad Software, La Jolla, CA).

Next, to exclude the possibility that the tested samples could reduce cell sensitivity to SARS-CoV-2, viral titers in untreated and C-POLAR-coated samples wash out solution were determined. Control and C-POLAR-coated 1 cm^2^ of nonwoven spunlace were washed in 10 mL of DMEM with 10% FBS by vortexing five times for 5 s. The 5 mL of wash-out solution was transferred to a new tube, and 50 µL of SARS-CoV-2 (strain hCoV-19/Czech Republic/NRL_6632_2/2020) was added and incubated for 30 min at room temperature. After incubation, 200 µL was removed, and the viral titer was determined by plaque assay in Vero E6 using 10-fold dilutions in duplicate. Briefly, a virus with cells (250,000 Vero E6 cells per one well in a 24-well plate) was gently mixed and incubated for 4 h at 37°C, 5% CO_2_ incubator. Then, 0.4 mL of 3% carboxymethylcellulose was added and incubated for 5 days at 37°C, 5% CO_2_. After incubation, the cells were washed once with 1× phosphate-buffered saline (PBS), stained with naphthol blue-black dye, washed with water, and air dried. Plaques were counted as plaque forming units per mL (pfu/mL), expressed in log_10_, compared to log_10_ pfu/mL of untreated control and test sample log_10_ differentials were determined.

### Effect of C-POLAR virucidal activity using viable SARS-CoV-2

To determine the effect of C-POLAR against SARS-CoV-2 activity, untreated and C-POLAR-coated materials were tested in triplicate, 50 µL of SARS-CoV-2 were dropped to 1 cm^2^ of nonwoven spunlace covered with glass coverslips and incubated for 5 and 30 min at room temperature in a six-well plate with a wetted cotton ball to preserve humidified conditions. Following the incubation, samples were transferred into 5 mL of DMEM with 2% FBS, vortexed five times for 5 s, and the virus titer was determined by plaque assay as above and expressed as pfu/mL. The SARS-CoV-2 titer reduction was expressed in percentage. To assess the statistical difference between untreated and C-POLAR-treated samples, a one-way analysis of variance (ANOVA) was performed (GraphPad Prism v.9.4.0). Results were considered statistically significant at *P* < 0.05.

### Preparation of bacterial strains

*S. aureus* (Reynold’s strain), *E. coli* (K-12), *Pseudomonas aeruginosa* (PA-01), and *Enterococcus faecalis* (clinical isolate) were grown from −80°C frozen stock in 4 mL of lysogeny broth (LB) medium overnight at 37°C in an orbital shaker. After overnight growth, bacterial cultures were subcultured by adding 1 mL of overnight growth into 3 mL of fresh LB broth and incubated for an additional 1 h, shaking at 37°C. About 2 mL of subcultured bacterial suspension was spun down at 1,000 × *g* for 2 min, after which the broth was aspirated, and the pelleted bacteria resuspended in 1 mL of PBS solution (Corning, Corning, NY).

### Bacterial inoculation of textile material

Untreated control, 4%, and 6% C-POLAR-coated textile samples were placed into a microfuge tube and soaked in PBS. The subcultured and washed bacterial suspension (50, 25, and 10 µL) were pipetted directly onto the control and test textile samples and incubated for 10 min at room temperature. About 450 µL of complete RPMI media (RPMI with 10% FBS, l-glutamine, without antibiotics) was added to the control and test samples in the microfuge tube. Media only served as a background control.

### Bacterial viability analysis

The viability of the bacteria from textile material was determined using PrestoBlue, a resazurin-based metabolic dye, and turbidity using OD_600_nm. For PrestoBlue, following inoculation and placement of fabric patches into microtubes, samples were incubated at 37°C, shaking for 1 h, after which 50 µL of PrestoBlue (ThermoFisher Scientific, Waltham, MA) was added. The tubes were incubated for 1 h, shaking at 37°C, and the color change was then detected on an i3X plate reader (Molecular Devices, San Jose, CA). For the OD_600_nm analysis, the inoculated fabric was placed in complete media and incubated at 37°C with shaking for 3 h. The OD_600_nm from a 100-µL media sample was performed on a plate reader to determine the relative turbidity. The viability of bacteria was calculated as the percent decrease in PrestoBlue or OD_600_nm using (treated/control*100)–100.

### Airborne removal of bovine coronavirus

Bovine coronavirus–laden aerosol removal studies followed the University of Minnesota Institutional Biosafety Committee (IBC) protocol number 1808-36316H. Particles were generated from a suspension of bovine coronaviruses in DMEM spiked with fluorescein dye prepared with a titer near 10^7^ TCID_50_ mL^−1^. We used a large particle-generating nebulizer operated with a syringe pump at 3 mL min^−1^, and SIMCO static control ionization source to bring particles to a steady-state charge distribution and minimize electrostatic effects. In earlier tests with this nebulization system, the size distribution of viable bovine coronavirus-carrying particles was determined and found to have a peak (mode) diameter near 2–3 μm and similar to the size distribution of particles produced via human respiratory activities.

The virus aerosol tests were run with filter face velocities of 0.51 m s^−1^ at room temperature (nominally 300K), and a relative humidity of ~30%. Upstream of the test filter, particles were sampled into an Andersen impactor (25) at a flow rate of 90 L min^−1^, with stages 0, 5, and 6 installed and a total filter after the impactor. Combining all stages, this setup led to the impactor collecting particles larger than ~200 nm in diameter and the filter collecting particles smaller than 200 nm in diameter.

To determine the efficacy of viral particle removal of bovine coronavirus, both virus titration, and RT-qPCR were determined to establish viable virus concentrations were determined for bovine coronavirus (26). Results are reported as average log reductions (LRs), calculated as: $LR=log_{10}\left(\frac{{\overset{-}{C}}_{up}}{{\overset{-}{C}}_{down}}\right)$ (2) where ${\overset{-}{C}}_{up}$ is the average upstream sampler concentration across all trials for either the titration or RT-qPCR test and ${\overset{-}{C}}_{down}$ is the average downstream concentration across all trials for either the titration or RT-qPCR test.

### Removal of airborne particles under passive conditions

To investigate the efficacy of C-POLAR at reducing airborne particles passively, a sealed chamber was created with a mixed ventilation system. The chamber (1.71 m × 1.76 m × 1.90 m) provided an isolated system where aerosolized particles could be generated and monitored with a controlled airflow. The ventilation system comprised an intake fan with an attached HEPA filter and an exhaust fan, with the inlet and exhaust vents inserted into the chamber’s ceiling. Ball valves were placed in the intake and exhaust ducts to further refine airflow control.

A compression nebulizer (Cool Mist Inhaler Compressor, Blue Echo Care) with a flow rate between 5 and 8 L/min generated a continuous stream of aerosol droplets from a solution. Sterile, non-neutralized 0.1 µm filtered PBS containing 1.06 mM KH₂PO₄, 154 mM NaCl, and 5.6 mM Na_2_HPO_4_ was the chosen nebulization solution. The nebulization solution provided floating salt particles. Particle concentrations were monitored throughout testing using a Fluke 985 Particle Counter, which detects and quantifies particles ranging between 0.3 and 10 µm in size. For all tests involving the interaction of C-POLAR with airborne particles, 6% C-POLAR-treated polyester nonwoven spunlace measuring 28 cm wide was used.

The aerosol source (nebulizer) was placed in the far-left corner of the chamber with the spout set at a height of 1.0 m, while the particle counter was placed in the opposite corner, 1.6 m away on a table of the same height. A box fan operating at 600 rpm was placed in the corner of the chamber to promote air circulation. The chamber ventilation settings comprised the inlet fan being turned off, and the inlet duct valve completely closed to prevent backflow. The exhaust fan ran on the lowest setting, measured at <0.5 air changes per hour (ACH) to create negative pressure within the chamber and ensure no particle leakage. Three different conditions were tested within the chamber: a control (*n* = 4), a single isolated curtain (*n* = 3), and wall hangings (*n* = 3). The C-POLAR curtain (1.0 m × 1.0 m) was placed perpendicular to and 60 cm from the nebulizer spout. Finally, the wall hangings consisted of ten 1.8 m long strips of C-POLAR-treated fabric hung on the walls and ceiling of the chamber, totaling 5.04 m^2^ and covering approximately 31% of the chamber interior surfaces.

Particle concentrations and decay time analysis were performed using a particle counter that collected 0.47 L air samples every 10 seconds throughout the 30-min test, measuring the concentration of 0.3–10 µm particles per liter. Each test began with 5 min of no nebulizer input to establish the baseline concentration of particles within the chamber. The nebulizer was turned on for 5 min, followed by a 20-min settling period. All particle concentration readings were normalized to the baseline concentration established in the first 5 min of each test. To analyze the reduction in particles, an exponential decay curve was fit to the final 15 min of settling time, and the decay time, *τ*, was calculated in minutes (17).

### Determining the efficacy of antibacterial and bacterial filtration efficiency over time

Bacterial filtration efficiency (BFE) was tested regarding the ASTM Standard Test Method F2101-19 (27), with some modifications. The C-POLAR-treated filter used in this study comprised 40 g nonwoven spunlace, which was conditioned for at least 4 h at 21 ± 5°C and relative humidity of 65 ± 5% before the BFE test. An aerosol spray of the challenging bacterium *S. aureus* in peptone water (approximately 5 × 10^5^ CFU/mL) was generated using a nebulizer (Rossmax NA100, Taipei, Taiwan). The sprayed aerosol (mean aerosol particle size ~2.2 µm) was then drawn through the test filter specimen (*n* = 3) under vacuum (with a flow rate of 100 L per minute) and collected on a tryptic soy agar (TSA) plate. The number of *S. aureus* colonies formed on the TSA plate was counted after incubated at 37 ± 2°C for 48 ± 4 h. Controls were conducted to evaluate the number of colonies formed on the TSA plate without the use of filter, where BFE was expressed in percent is calculated by the following equation:


(3) $$\%BFE=\;\frac{C-T}{C}$$

*C* and *T* are the total average plate count for the controls and test filters, respectively.

To determine any change to BFE of the C-POLAR-treated filter caused by aging, accelerated aging was conducted in accordance with the ASTM Standard Test Method F1980-21 (28) to expose the C-POLAR-treated under elevated temperature conditions, with an assumption to the use of default conservative aging factor of 2.0. The C-POLAR-treated filter and a control filter without C-POLAR treatment were incubated at a test temperature of 120 ± 2°C for 48 h, equivalent to an estimated aging of 5 years.

Antibacterial property after accelerated aging was conducted following the ASTM International Standard F1980-19 (28) to expose the C-POLAR-treated filter under elevated temperature conditions, assuming the default conservative aging factor of 2.0. The C-POLAR-treated filter was incubated at a test temperature of 60 ± 2°C, where a specimen was collected from the incubator on 0 (control), 8, 16, 32, and 64 days to an ambient temperature at 22 ± 3°C before the study of antibacterial activity. Under this condition, aged specimens with 8, 16, 32, and 64 days were equivalent to 0.31, 0.62, 1.2, and 2.5 years, respectively.

To determine the antibacterial activity study of the aged filter samples, the International Organizational of Standardization standard ISO 20743: 103 (29) was used. Briefly, test inoculum of bacterial cultures of *S. aureus*, *E. coli*, and *P. aeruginosa* were streaked separately onto plates with TSA at 37 ± 2°C for 24 h A colony from the resulting plate was transferred to peptone-salt solution by an inoculating loop, with the bacterial concentration measured by spectrophotometry at 600 nm. The number of bacteria was diluted to a concentration of 1–3  ×  10^6^ CFU/mL using peptone-salt solution. About 1 mL of resulting inoculum was added to a plate with agar and spread evenly using an inoculation spreader.

Both the filter specimens before and after accelerated aging were cut in circular shapes (*n* = 6 for each bacterial species) with a diameter of 3.8 cm. As required by ISO 20743, a fabric material made of cotton without any antimicrobial treatment was used as a control to compare the antibacterial activity of the aged filters. Each cut specimen was put onto the agar surface of bacterium pre-inoculated plate with a 200 g stainless steel cylinder weighed down for 60 ± 5 s to facilitate the transfer of the bacterial cells. Immediate after the transfer, half of the specimens (i.e., *n* = 3) were placed individually into a stomacher bag with 20 mL of neutralizing solution (composed of 30 g polysorbate 80, 3 g egg-yolk lecithin, 1 g histidine hydrochloride, 1 g meat or casein peptone, 4.3 g sodium chloride, 3.6 g monopotassium phosphate, 7.2 g disodium phosphate dihydrate and makeup to 1,000 mL) to shake out the bacterial cells using a stomacher. About 1 mL of each of the shake-out solution and its dilution series (1:9 for each dilution by peptone-salt solution) were added to a plate with TSA. The plates were incubated for 24 h at 37 ± 2°C. The remaining half of the specimens were incubated in an incubator for 24 h at 37 ± 2°C and conducted the above shake-out, inoculation to TSA, and incubation processes. After incubation, the number of colonies on the plates was enumerated. The antibacterial activity value (in logarithm scale) was calculated by the following equation:


$$Antibacterial\;activity\;value=\;\left(\log_{10}C_{t}-\log_{10}C_{0}\right)-\left(\log_{10}T_{t}-\log_{10}T_{0}\right)$$

where *C_t_* is the arithmetic average of the number of bacteria from the shake-out solution of the control specimens after 24-h incubation; *C_0_* is the arithmetic average of the number of bacteria from the shake-out solution of the control specimens immediately transfer; *T_t_* is the arithmetic average of the number of bacteria from the shake-out solution of the aged filter specimens after 24-h incubation; and *T_0_* is the arithmetic average of the number of bacteria from the shake-out solution of the aged filter specimens immediately transfer.

### Statistical analysis

Microsoft Excel (Microsoft 365 MSO, version 2202) was used for the statistical analysis. A *t* test or analysis of variance (ANOVA) for non-parametric data using GraphPad Prism (version 9.4.0) and a *P* value < 0.05 was considered significant.

## RESULTS

### C-POLAR inactivates viable SARS-CoV-2

**Fig 1:**
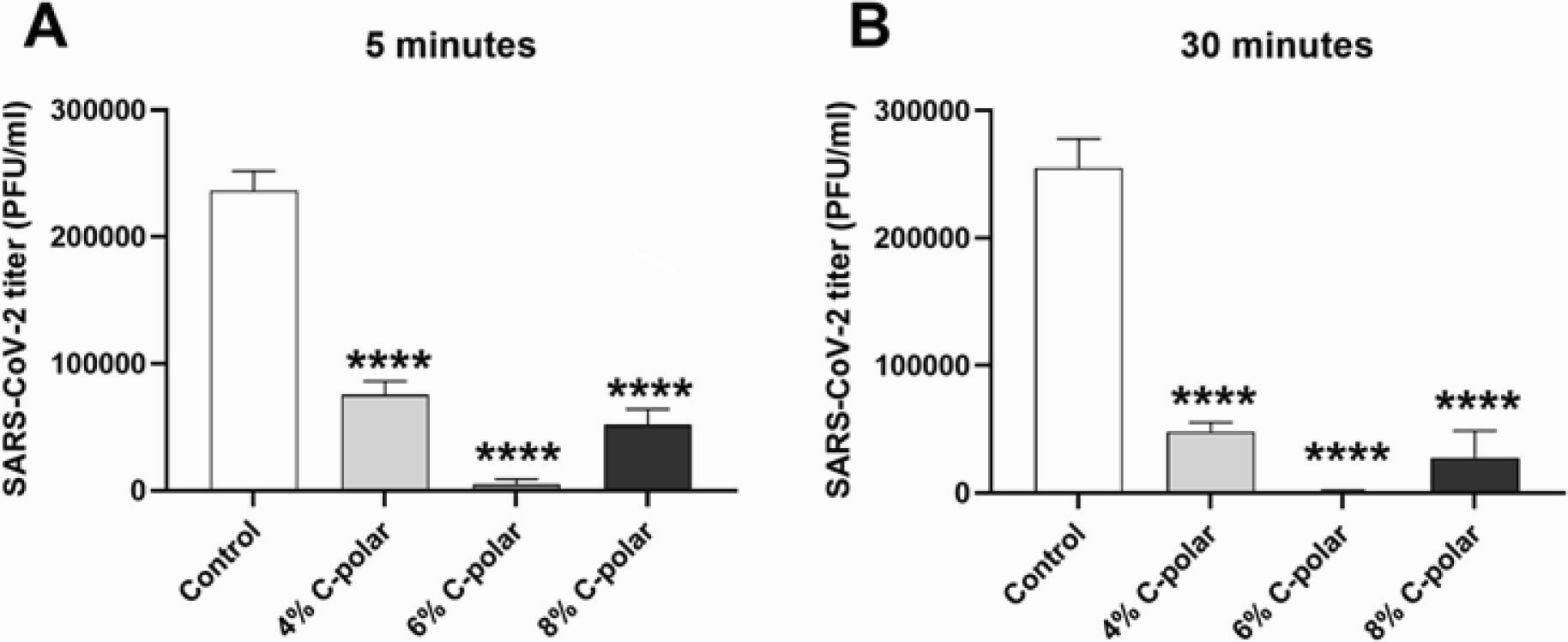
C-POLAR-treated textile eliminates SARS-CoV-2 virus. Viral titer after contact with control (untreated), 4%, 6%, and 8% C-POLAR-treated cellulose/polyester-based textile for 5 min (**A**) and 30 min (**B**). SARS-CoV-2 titers were determined in Vero E6 cells and expressed as PFU/mL. Values are depicted as mean and standard deviation from three biological replicates. Asterisks indicate statistically significant differences between the control (untreated) and C-POLAR-treated-textile determined by one-way analysis of variance (ANOVA), ****P < 0.0001.

Using untreated and C-POLAR-coated 1 cm^2^ of 40 g nonwoven spunlace, the C-POLAR anti-SARS-CoV-2 activity was determined according to the Standard ISO 18184-2019-06. Three concentrations of C-POLAR (4%, 6%, and 8%) were tested. The anti-SARS-CoV-2 activity of 4%, 6%, and 8% C-POLAR-treated nonwoven spunlace was determined for 5- and 30-min contact time (Table 1). The 5-min exposure led to a 98% reduction of SARS-CoV-2 titer (1.66 log pfu/mL reduction, Fig. 1, panel A). Extending the exposure to 30 min reduced the SARS-CoV-2 titer by 2.4 log pfu/mL (Fig. 1, panel B). Interestingly, the 6% concentration of C-POLAR was optimal for anti-SARS-CoV-2 inactivation.

**TABLE 1:** Anti-SARS-CoV-2 activity of C-POLAR-treated spunlace

C-POLAR concentration (wt/wt%)	Contact time (min)	Cellulose/polyester-based textile	
		SARS-CoV-2 titer reduction (%)^*a*^	*P* value^*b*^
4%	5	68 ± 4.6	<0.0001
	30	81 ± 2.8	<0.0001
6%	5	98 ± 1.6	<0.0001
	30	99.6 ± 0.004	<0.0001
8%	5	78 ± 5.2	<0.0001
	30	89 ± 8.3	<0.0001

^*a*^ SARS-CoV-2 titer reduction was calculated from average SAR-S-CoV-2 titers from untreated textile set as 100% minus the quotient between averages from SARS-CoV-2 titers from C-POLAR-treated and untreated textiles. Results are reported as mean and standard deviation expressed in percentage from three biological replicates.

^*b*^ One-way analysis of variance test between SARS-CoV-2 titers determined from untreated versus C-POLAR-treated textiles at given contact time.

To determine if direct toxicity of the C-POLAR to cells as a possible confounder, the XTT cell proliferation and viability assay in Vero E6 and nasopharyngeal cells was performed. The wash-out solution from the C-POLAR-treated 40 g nonwoven spunlace composed of 50% Polyester/50% Cotton exhibited only moderate toxic effect in Vero E6 cells in the highest dilution (Fig. S1) or nasopharyngeal cells (Fig. S2). As a further control, the possibility that C-POLAR can reduce cell sensitivity to the virus was determined. Here, the same amount of SARS-CoV-2 was added to wash out the solution from each control (untreated), 4%, 6%, and 8% C-POLAR-treated nonwoven spunlace virus titer was determined by plaque assay. The comparison between SARS-CoV-2 titers from untreated control wash-out and wash-out solution from C-POLAR-treated nonwoven spunlace showed a 0.02–0.14 log pfu/mL difference, which is well below the 0.5 log pfu/mL limit indicated by the ISO Standard.

### Removal of beta-coronavirus using an airborne dissemination model

**Fig 2:**
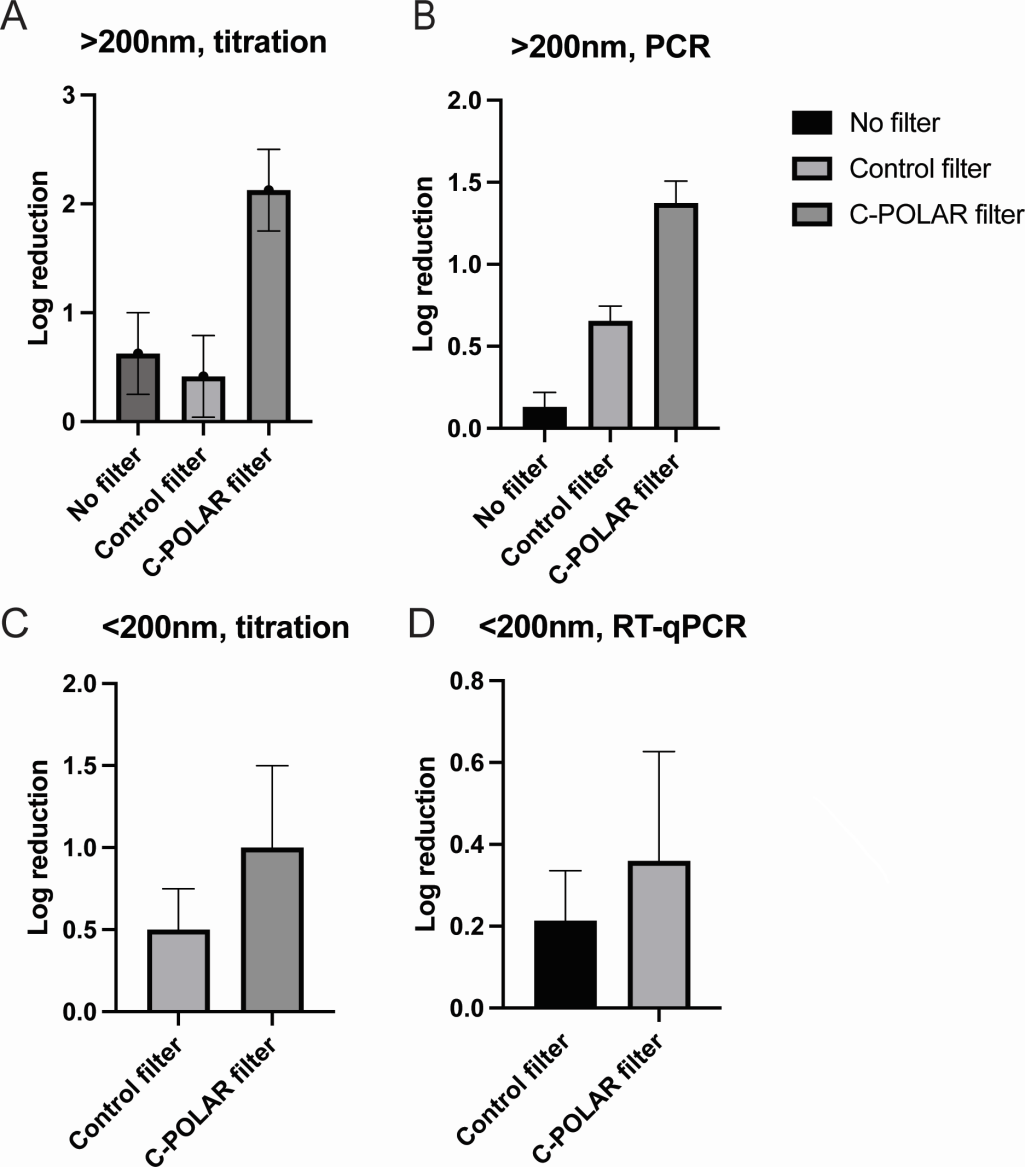
Removal of aerosolized bovine coronavirus in a velocity wind tunnel. The log reduction (base 10) in bovine coronavirus aerosol concentration after passing through filters at a face velocity of 0.51 m s^−1^. Log reduction is based upon virus titration, for impactor sampled particles (larger than ~200 nm in diameter) (A). RT-qPCR, for impactor sampled particles (larger than ~200 nm in diameter) (B). Virus titration, for virus recovered from a total filter (smaller than 200 nm in diameter) (C). RT-qPCR, for virus recovered from a total filter (D).

To determine the effectiveness of C-POLAR-treated filters using conventional models of HVAC systems, the removal of bovine coronavirus particles was determined. Airflow tests were performed either (i) without any filter (positive control), (ii) with the control filter, or (iii) with the C-POLAR filter. Bovine coronavirus aerosolization and sampling were carried out for two to three 30-min periods with each of the three noted test configurations (no filter: two trials; control filter: three trials; and CPS filter: two trials). The test filters had visibly collected particles, and particle deposition occurred on the Andersen impactor stages; this observation was made evident by adding fluorescein dye to the particles. Pooled samples were extracted from stages 0, 5, and 6 for each impactor, with a separate sample for the total filter, following previously described methods (30). Based on RT-qPCR and titration, 0.5–1.5 log reductions were calculated (Fig. 2, panels A through D).

### Removal of airborne particles in a passive manner

**Fig 3:**
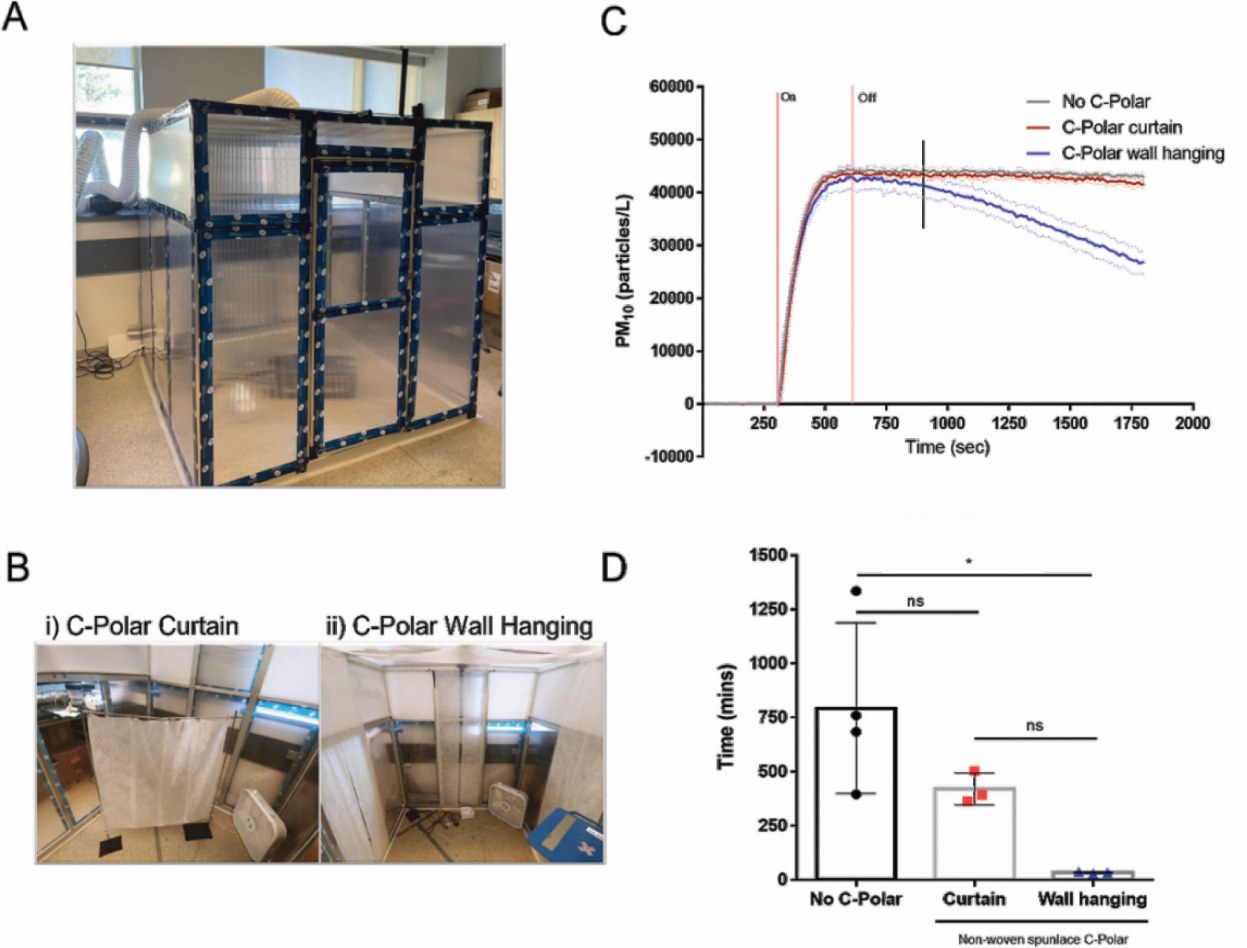
Impact of C-POLAR static wall hanging on passive on airborne particles in an enclosed chamber. An enclosed clean chamber was constructed (1.71 m × 1.76 m × 1.90 m) with mixing ventilation (A). Mixing ventilation for the clean chamber (<0.5 ACH), (B, Photo i.) Single layer 6% C-POLAR-treated nonwoven spunlace curtain design (total surface area: 1.0 m^2^) (B, Photo ii.) Single layer 6% C-POLAR-treated nonwoven spunlace wall-hanging design and testing locations (total surface area: 5.04 m^2^). Average airborne particle concentrations normalized to their respective 5 min baseline average (PM10 = particles between 10 µm and 0.3 µm diameter) (SD = dotted line) (C). Particle source = PBS (154 mM NaCl). Nebulization time = 5 min (shown between two vertical red lines). Vertical black line indicates when decay was fitted. Calculated decay time determined by exponential curve fit (D). One-way ANOVA with Tukey’s multiple comparisons, *α* = 0.05.

The effectiveness of C-POLAR-treated textile in the passive reduction of airborne particles was explored by measuring particle reduction during settling time. Particle concentrations during settling times were fitted with exponential curves to determine decay time. The enclosed chamber tests revealed that integrating C-POLAR wall hangings into a room environment can significantly reduce the decay time of airborne particles compared to control. The decay time (*τ*) is determined by the exponential curve model *A = A_0_e^−kt^*, where *τ* equals the inverse of the rate constant *k* (31). A shorter decay time indicates a faster particle clearance rate by the presence of a sink (e.g., HVAC, portable air filter, and surface deposition) (32–35). A C-POLAR curtain did not significantly reduce airborne particles compared with control. However, the reduction became significant when the effective surface area was increased fivefold with wall hangings (Fig. 3).

The maximum particle concentration is defined as the highest value of particles recorded during each test (Table 2). Values were similar for all conditions at approximately 44,000 particles/L. All tests reached consistent decay rates in the last 15 min of the settling period and were fitted with an exponential curve accordingly (Table 2). The decay time, *τ*, was calculated for each condition, with a mean value of 794 ± 341 min for no C-POLAR, 420 ± 60 min for the curtain, and 34 ± 3.0 min for the wall hangings. A one-way ANOVA with Tukey’s *post hoc* testing revealed a significant reduction in the decay time for airborne particles between the control without C-POLAR and wall hangings conditions.

**TABLE 2:** Summary of experiments; description, maximum particle concentration data normalized to baseline concentration, and decay time (*τ*) values

Experiment number	Description	Maximum particle concentration (#/L)	*τ* (min)
1	Control	45,805.9	396.0
2	Control	45,066.3	760.5
3	Control	44,616.2	1,335.1
4	Control	44,304.9	684.5
5	Curtain	44,003.4	503.3
6	Curtain	45,037.3	393.7
7	Curtain	43,456.3	363.5
8	Wall hangings	42,269.3	30.3
9	Wall hangings	41,974.7	34.1
10	Wall hangings	45,771.9	37.6

### C-POLAR-treated material reduces Gram-positive and Gram-negative viability

**Fig 4:**
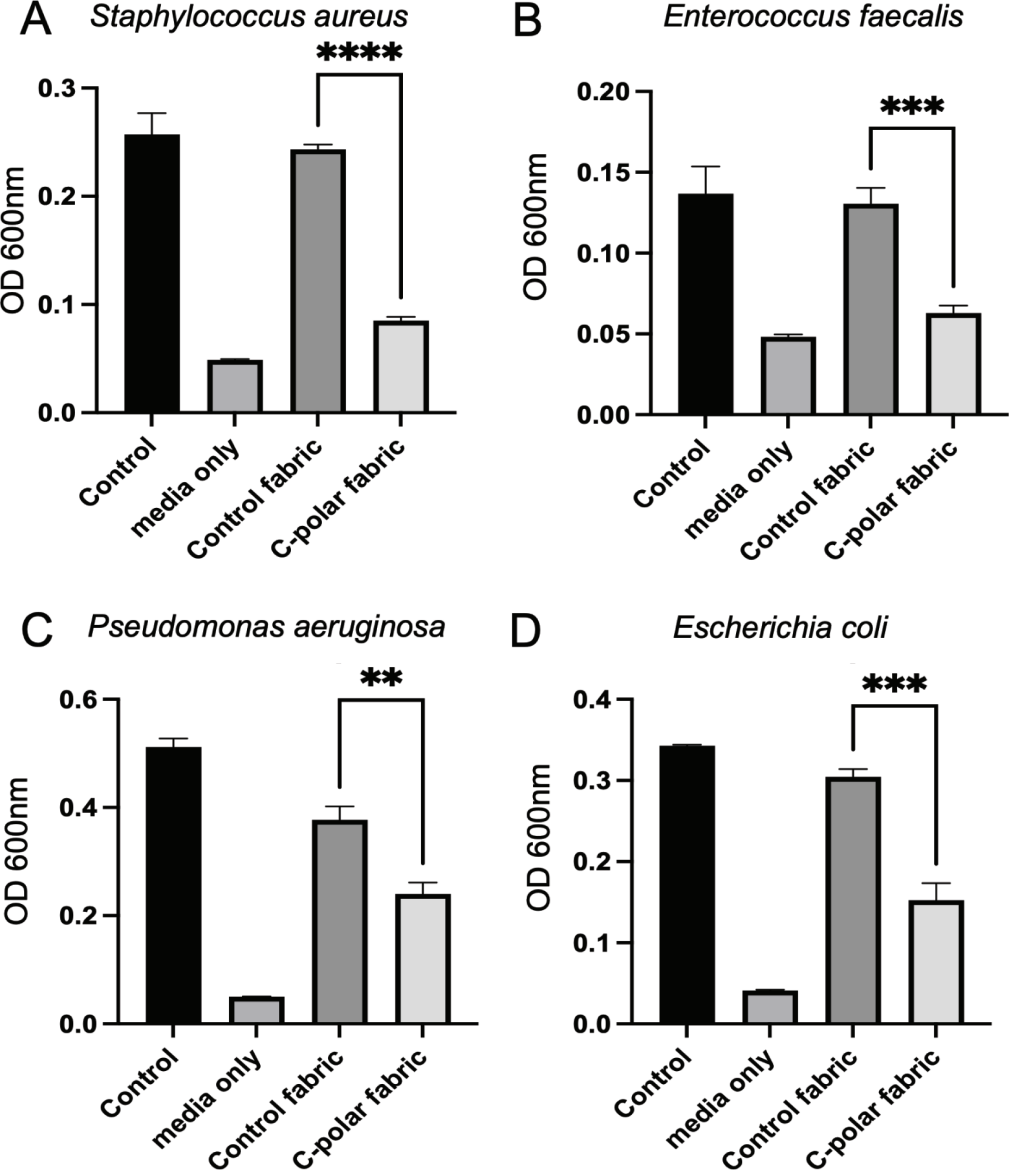
Effect of C-POLAR on the viability of bacterial pathogens. Gram-positive (*Staphylococcus aureus* and *Enterococcus faecalis*) or Gram-negative (*Pseudomonas aeruginosa* and *Escherichia coli*) were directly inoculated into media (control), control fabric, or 6% C-POLAR-coated textile, incubated for 10 min at room temperature and then 450 µL of complete RPMI media (RPMI with 10% FBS, l-glutamine, without antibiotics) was added to the fabric patches in the same microfuge tube. Media only (no textile or bacteria) served as a background control. The inoculated textile was incubated by shaking at 37°C for 3 h, at which point the turbidity was measured from the suspension at OD_600_nm. Asterisks indicate statistically significant differences between the control (untreated) and C-POLAR-treated-textile determined by a two-tailed *t* test, **P < 0.02, ***P < 0.004, and ****P < 0.0001.

We next tested if positively charged textile surfaces are capable of intervening on the contact spread of bacterial pathogens. The effect of C-POLAR-treated textiles on the viability of Gram-positive bacteria, *S. aureus*, and *E. faecalis* was determined. Compared to control textiles, materials treated with C-POLAR demonstrated a significant decrease in *S. aureus* viability using Prestoblue as a readout of viable metabolic activity. Textiles treated with 4% or 6% C-POLAR resulted in a 90% and 92% reduction of viability, respectively, compared to the control (*P* value < 0.0001, for both). No significant difference was observed between the 4% and 6% treated groups (*P* value > 0.05). OD_600_nm revealed a significant *S. aureus* growth reduction of 65% following inoculation of C-POLAR-treated nonwoven spunlace compared to untreated control (*P* value < 0.0001) (Fig. 4). Similar studies with *E. faecalis* show comparable significant reductions in bacterial viability of 52% between treated and untreated material (*P* value = 0.0002) (Fig. 4, panels A and B).

To test if the textile amount could influence the viability of inoculated bacteria, thicker, 145 g woven fabric was used in viability assays. Thick textile material showed a persistent reduction in Gram-positive and Gram-negative bacterial viability with 6% C-POLAR coating, resulting in an 83% viability reduction compared to the control (*P* value < 0.0001).

We next determined the effect of the C-POLAR-coated material on the Gram-negative pathogens *E. coli* and *P. aeruginosa*. Compared to the control material, the C-POLAR-containing textiles significantly reduced *P. aeruginosa* viability by 36% (*P* value = 0.0009) (Fig. 4, Panel C). Similar effects were observed with *E. coli*. Control and treated material were inoculated with *E. coli* with a 50% loss of viability (*P* value = 0.0001). Thicker treated textiles (e.g., 145 g woven fabric) show a similar reduction in viability compared to the control of viability of 36% (*P* value < 0.0001) (Fig. 4, Panel D).

### BFE following accelerated aging

**Fig 5:**
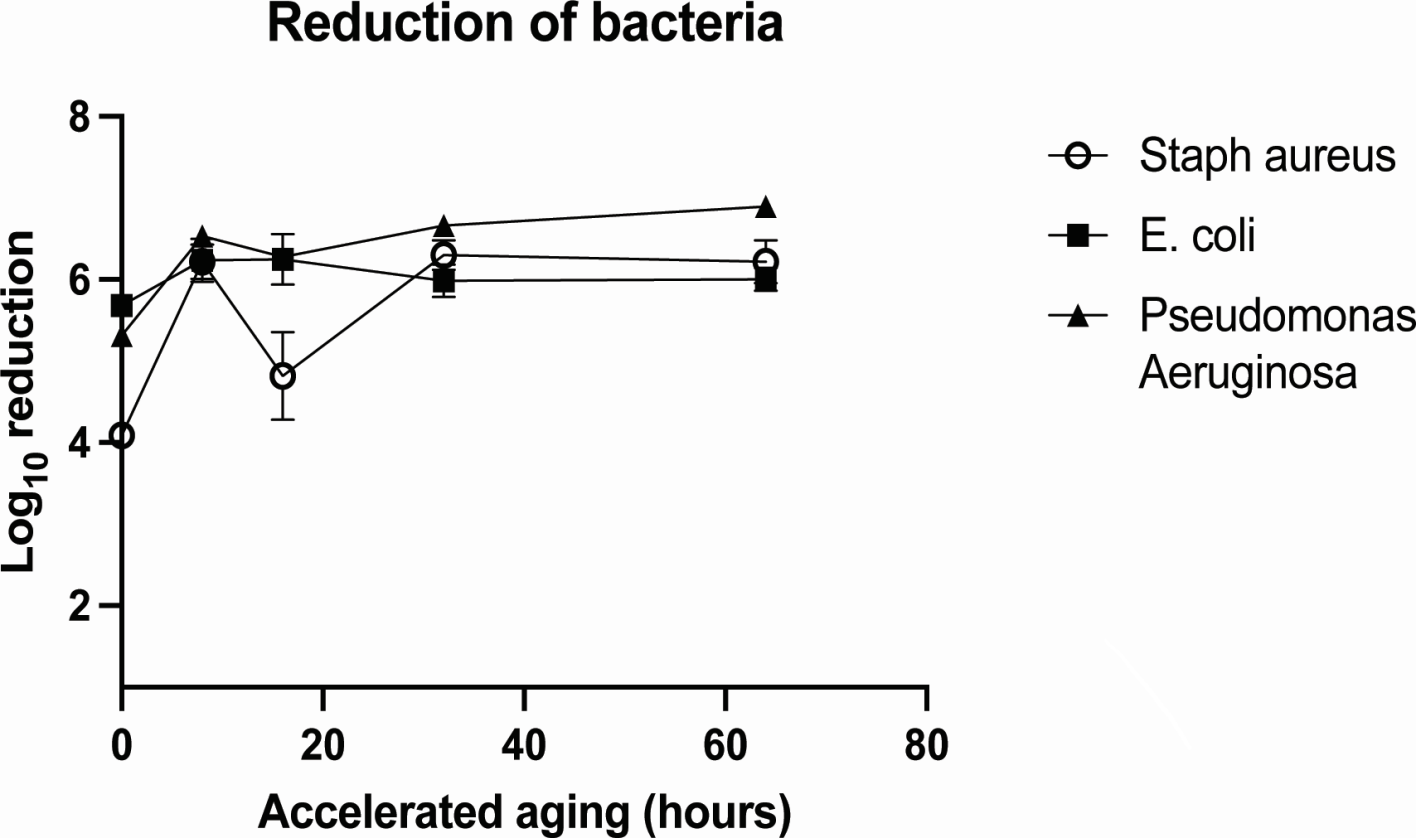
Impact of accelerated aging of C- POLAR-treated filters on bactericidal effect. C-POLAR-treated filters were subjected to accelerated aging by exposure to temperature of 60 ± 2°C for a period of 64 days. After different days of accelerated aging, C-POLAR textiles were inoculated with different bacterial pathogens, *Staphylococcus aureus*, *Escherichia coli*, and *Pseudomonas aeruginosa* and viability determined.

To determine the stability of the C-POLAR-treated filter to inactivate pathogens over time, an accelerated aging study of the filter was performed, and *S. aureus* was used as the testing species. The BFE of the untreated filter (control) was found to be 94.3 ± 1.8%, where both the C-POLAR-treated filters before and after aging arrested all bacterial colonies (with BFE 100.0 ± 0.0%) in aerosol under the tested conditions and were found to be significantly different to the control (Fig. 5). The BFE of the C-POLAR-treated filter before and after accelerated aging was comparable to other filter materials, such as electrospun polylactic acid nanofibers (18). The antibacterial activity of the C-POLAR-treated filter on the viability of Gram-positive bacterium (*S. aureus*) and Gram-negative bacteria (*E. coli* and *P. aeruginosa*) was determined before and after a prolonged period of accelerated aging. Antibacterial activities of the C-POLAR-treated filter after 64-day accelerated aging (equivalent to an aging of 2.5 years under the default conservative aging factor of 2.0), where the antibacterial activities were maintained between 4.09 and 6.89 (>99.99% reduction) for all three bacterial species throughout the period of accelerated aging (Fig. 5). No decreasing trend of antibacterial activity was noted during the accelerated aging period.

### On-site durability study

A C-POLAR-treated filter was installed on-site in an air handling unit in the International Commerce Centre of Hong Kong to mimic a real-life air circulation environment. After a 1-month operation, the used filter was removed to assess the antibacterial properties per ISO 20743:2013 (29), using *S. aureus* as the testing species. No bacterial colony was identified from the shake-out solution after 24-h incubation, and the reduction of bacterial activity of the used filter was found to be 5.72 (in log10 scale) lower compared to the control. The performance in antibacterial properties in the aged filter was comparable to a newly coated C-POLAR-treated filter.

## DISCUSSION

Our study is the first to examine the cationic C-POLAR-treated material and its potential use in preventing viral and bacterial viability and transmission. These data demonstrate that a cationic polymer such as C-POLAR-treated material can capture and reduce the viability of various pathogens, including viruses and Gram-positive and Gram-negative bacteria.

Despite being enveloped, coronavirus possesses negative potential molecules related to lipid molecules, amino acid composition, and spike proteins (36). In air filtration tests, the C-POLAR-treated material was able to inactivate coronaviruses, it is known that bovine coronavirus particles fall into the micrometer size range. The C-POLAR filter demonstrated a high removal efficiency, leading to increased log reduction of virus. Meanwhile, the control filter collection efficiency was below 0.50 near 1 µm, insufficiently reducing virus concentrations. For sub-200 nm particles collected on the total filters of samplers, an increase in log reduction is also observed for the C-POLAR filter with the CPS filter titration-based log reduction near 1.0 (90% removal). Bovine coronavirus aerosol testing is consistent with physical particle collection efficiency testing, demonstrating that electret coatings can be incorporated into filter composites with lower-grade filters as their base. These composite filters can have high log reductions for virus-laden aerosols.

We also turned attention to pathogens spread via fomite or contact. Previous literature suggests a high contamination rate of white coats and scrubs with different bacterial pathogens such as *S. aureus* (37). Antibiotic-resistant bacteria such as methicillin-resistant *S. aureus* can also be found at a high colonization rate on white coats of healthcare providers (38, 39), highlighting the likely chain of transmission by fomites in the hospital environment to patients.

A primary focus on prevention was associated with handwashing and sanitization of hospital equipment (39–41). However, with the persistence of high contamination rates, recent studies have focused on the laundering and use of antimicrobial-coated gowns to break the chain of contact transmission and improve healthcare safety. Hospital-laundered and disposable scrubs show less contamination as compared to home-laundered and unwashed scrubs. Clinical testing of antimicrobial-coated fabrics did not limit the growth of Gram-positive and Gram-negative bacteria (42, 43). Specific charged coatings, such as charged chitosan, on hospital scrubs did not show a significant difference for *S. aureus*, *Enterococcus* spp., and Gram-negative rods compared to untreated scrubs (*P* value = 0.50, 0.17, and 0.55, respectively). On the other hand, our study demonstrated a significant difference in viability with C-POLAR-treated material for all types of bacteria (*P* value < 0.05 for all) (44). Interestingly, specific antimicrobial activities depended on the species, which may reflect the net charge based on the composition of the cell wall (45, 46). Other studies showed similar results for different antimicrobial agents tested and limited to no protection against Gram-negative and Gram-positive bacteria (38).

Our data demonstrate that C-POLAR-treated material retains activity despite accelerated aging mimicking 5 years. In addition to conventional air filtrations, C-POLAR-treated materials could clear airborne particles using static hangings as measured by rapid particle decay. The findings confirm that C-POLAR wall hangings can be a significant sink for airborne particles when HVAC parameters cannot be considered (47). This finding is consistent with our understanding that increasing surface area allows more significant interaction between airborne particles and the porous cationic nonwoven spunlace (48, 49). Future investigations must explore C-POLAR in passive and active filtration solutions to reduce airborne particle concentrations.

Our findings have several limitations, including a need for clinical studies on nosocomial infections in hospital or healthcare settings. Future investigations will be essential to determine the antibacterial effect of the C-POLAR coating on antibiotic-resistant species and other pathogens, such as invasive fungal pathogens and more expansive nosocomial pathogens. Finally, controlled clinical research trials would be ideally used to study the real-life impact of such technologies.

Our study demonstrates the broad application and utility of a cationic polymer such as C-POLAR-treated material that can be used as a mitigation strategy for airborne and/or contact transmission of viruses or bacterial species. By applying cationic polymer technologies to conventional air filtration and textiles, the quality of the environment can be improved, allowing for an infection resilient for the improved health status of at-risk populations.

## SUPPLEMENTAL MATERIAL

Fig. S1 spectrum.04097-23-s0001.pdf Vero cell cytotoxicity studies.

Fig. S2 spectrum.04097-23-s0002.pdf NP69 cell cytotoxicity studies.

Supplemental material spectrum.04097-23-s0003.tif Graphical abstract.

ASM does not own the copyrights to Supplemental Material that may be linked to, or accessed through, an article. The authors have granted ASM a non-exclusive, world-wide license to publish the Supplemental Material files. Please contact the corresponding author directly for reuse.
