# Supplementary material for: Effect of multifunctional cationic polymer coatings on mitigation of broad microbial pathogens
Source: Microbiol Spectr. 2024 Aug 5;12(9):e04097-23. doi: 10.1128/spectrum.04097-23 (PMC11370243; doi:10.1128/spectrum.04097-23)
Supplement: Fig. S1 — Vero cell cytotoxicity studies. [file spectrum.04097-23-s0001.pdf]

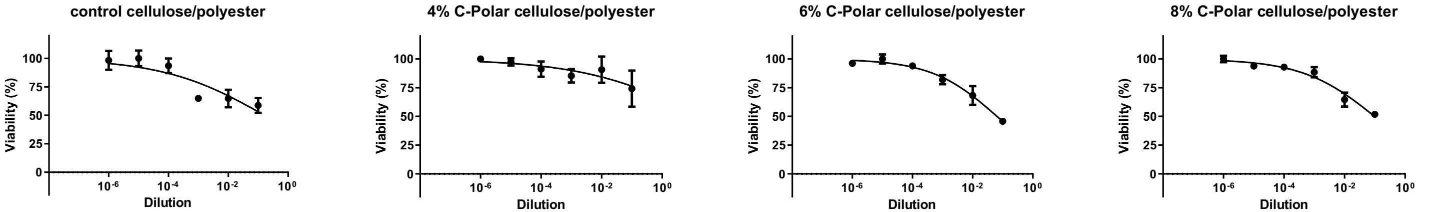

Supplemental Figure 1. Cytotoxicity of control (untreated) and C-Polar treated textiles on Vero E6 cells. Cytotoxicity of control (untreated), 4%-, 6%-, and 8%-C-Polar treated cellulose/polyester-based textile was expressed as a percentage of cell viability at the indicated dilution of wash-out solution.
