# Supplementary material for: Effect of multifunctional cationic polymer coatings on mitigation of broad microbial pathogens
Source: Microbiol Spectr. 2024 Aug 5;12(9):e04097-23. doi: 10.1128/spectrum.04097-23 (PMC11370243; doi:10.1128/spectrum.04097-23)
Supplement: Fig. S2 — NP69 cell cytotoxicity studies. [file spectrum.04097-23-s0002.pdf]

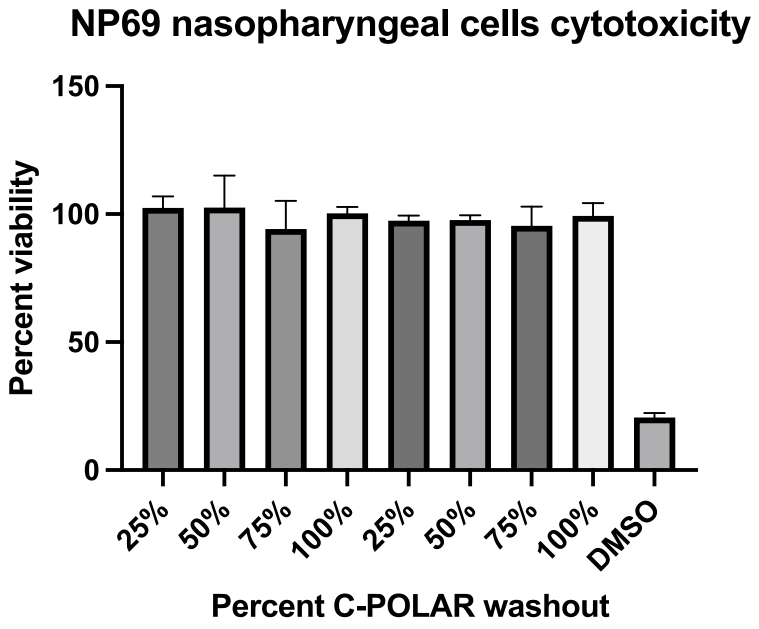

Supplemental Figure 2. NP69 normal nasopharyngeal epithelial cell viability in the presence of C-POLAR extracts. Following incubation with the different concentrations of extractable substances from the C-POLAR and blank (without coating) filters. A positive control (i.e. 20% DMSO) was used to illustrate cytotoxic effect to the NP69 cells.
